# Editorial: Neuro-immune interaction in disease

**DOI:** 10.3389/fncel.2026.1931155

**Published:** 2026-07-29

**Authors:** Na Sun, Marta Celorrio, Yiying Zhang, Xiaoying Chen

**Affiliations:** 1 Whitehead Institute for Biomedical Research, Cambridge, MA, United States; 2 Pediatrics Department, Washington University in St. Louis, St. Louis, MO, United States; 3 Department of Anesthesia, Critical Care and Pain Medicine, Massachusetts General Hospital and Harvard Medical School, Boston, MA, United States; 4 Laboratory of System Neuroimmunology, Institute for Immunology, Tsinghua University, Beijing, China; 5 IDG/McGovern Institute for Brain Research, Tsinghua University, Beijing, China; 6 Tsinghua-Peking Center for Life Science, Beijing, China; 7 School of Basic Medical Sciences, Tsinghua Medicine, Tsinghua University, Beijing, China

**Keywords:** Alzheimer's disease, microglia, neuroimmune interaction, neuroinflammation, Parkinson's disease, post-ischemic neurodegeneration, T cells

The recognition that the central nervous system (CNS) and the immune system are not isolated compartments but partners in a continuous dialogue has reshaped how we understand neurological disease. What was once framed as the brain's “immune privilege” is now understood as a tightly regulated, bidirectional exchange that sustains homeostasis under healthy conditions and, when dysregulated, becomes a driver of pathology. Microglia, once thought quiescent, continuously survey the parenchyma and react within minutes to perturbation (Nimmerjahn et al., 2005), and the discovery of functional lymphatic vessels in the dural sinuses revised long-held assumptions about how immune cells enter and leave the CNS (Louveau et al., 2015). The Research Topic, “*Neuro-immune interaction in disease*,” brings together five contributions, including two original studies and three reviews, that map this territory across human postmortem tissue, animal models, and emerging molecular mechanisms. Spanning Alzheimer's disease, Parkinson's disease, and post-ischemic neurodegeneration, the collection illustrates both how much the neuroimmune framework now explains and how much remains contested.

Several threads run through these papers. The first is the centrality of communication between neurons, glia, and peripheral immune cells, a principle now well established across neurodegenerative disorders, where misfolded proteins engage glial pattern-recognition receptors and trigger an innate immune response that shapes disease progression (Heneka et al., 2015). Chu et al. provide a striking example in human Parkinson's disease and related disorders. Examining postmortem substantia nigra across control, prodromal, Parkinson's disease, and progressive supranuclear palsy cases, they show that CX3CL1, a chemokine that mediates neuron-to-microglia signaling, is reduced in surviving neurons but elevated in vascular endothelial cells. The decline in neuronal fractalkine tracked with microglial density, while its rise in endothelium correlated with infiltrating CD4^+^ T cells. Notably, synucleinopathy and tauopathy displayed a shared pattern, suggesting that this dual shift is a convergent feature of neuroinflammation rather than a disease-specific one. This places a single signaling axis at the intersection of resident glial activation and peripheral immune recruitment, consistent with the broader recognition that both central and peripheral immune compartments are altered in Parkinson's disease (Roodveldt et al., 2024).

A second theme is that neuroimmune signaling extends well beyond the brain's borders. Müller and Di Benedetto surveyed the cellular and molecular foundations of neuroimmune crosstalk, with particular attention to Alzheimer's disease. Their mini-review situates microglial activation and cytokine signaling within a wider physiology that includes the blood–brain barrier, the glymphatic system, circadian regulation, and the gut-brain axis. By framing dysbiosis, barrier integrity, and sleep as modulators of neuroinflammation, they argue for integrative therapeutic strategies, targeting immune modulation, microbiota, and circadian alignment together rather than in isolation. This aligns with growing evidence that gut microbial dysbiosis can promote systemic chronic inflammation, compromise barrier integrity, and thereby influence neurodegeneration and brain aging (Mou et al., 2022).

The molecular machinery of this interface is the focus of Pluta, who reviews how non-coding RNAs act in concert with amyloid and tau to shape neuroinflammation in the post-ischemic brain. Here the dual nature of the inflammatory response comes to the fore: in the hours after ischemia, inflammation supports repair and homeostasis, but when it becomes chronic and uncontrolled it inflicts secondary damage and accelerates progression toward Alzheimer's-type dementia, a disequilibrium between protective and destructive immune programs that is increasingly seen as central to ischemic brain injury (Petrovic-Djergovic et al., 2016). By examining how microRNAs, circular RNAs, and long non-coding RNAs may reinforce a self-perpetuating cycle with protein aggregation, the review highlights regulatory layers that could prove tractable as therapeutic targets and clarifies why post-ischemic neurodegeneration so often converges with classic neurodegenerative pathology (Pluta).

The remaining two contributions confront a question the field cannot yet answer with confidence: how prominent, and how generalizable, is the peripheral immune presence within the diseased brain? Datta et al. make the case for the aging rhesus macaque as a translational model that captures features mouse models miss. Because macaques possess human-like association cortices, naturally carry ApoE-ε4, and spontaneously develop tau and amyloid pathology, they permit study of early, soluble hyperphosphorylated tau species, including pT217Tau, that degrade too rapidly to observe in human tissue. Their synthesis points to age-related inflammatory signaling as an upstream driver of calcium dysregulation, which in turn promotes tau phosphorylation and amyloid accumulation, and reports that anti-inflammatory and calcium-modulating interventions can reduce pathology. The argument is explicitly preventive: identifying the earliest inflammatory triggers, in a model that reflects primate immunology, may open therapeutic windows before pathology becomes entrenched.

Set against this, Oxendine et al. inject a valuable note of caution. Using single-nucleus RNA sequencing from ROSMAP, the largest available postmortem Alzheimer's cohort, they find no significant increase in parenchymal T cells in the prefrontal cortex or hippocampus, contrary to several prior reports, including evidence of clonally expanded CD8^+^ T cells patrolling the cerebrospinal fluid in Alzheimer's disease (Gate et al., 2020), and replicate the null result in dorsolateral prefrontal cortex using the SEA-AD atlas. Where they could detect the previously reported T cell expansion in middle temporal gyrus, its strength depended on donor age. Their conclusion is that T cell enrichment may be regionally restricted rather than widespread, and that brain region, analytical method, and dataset composition heavily shape what investigators find.

Read together, Datta et al. and Oxendine et al. expose a productive tension. One argues that inflammation is an early and causal force in sporadic Alzheimer's disease; the other shows that one of the most-cited markers of immune involvement/parenchymal T cell infiltration may be more limited and more methodologically contingent than assumed. These positions are complementary rather than contradictory: they jointly demand that claims about neuroimmune involvement be specified by region, cell type, disease stage, and model system. This insistence on specificity is the collective contribution of the Topic. Neuroimmune interaction is real and consequential, but its details including which signal, which cell, which compartment, which moment in the disease course, determine whether it is protective or destructive.

Several open questions follow. How can findings from human postmortem tissue, which captures end-stage pathology, be reconciled with model systems that access earlier, more dynamic phases? Which neuroimmune signals are causal drivers vs. downstream consequences? And how should the field standardize regional sampling and analytical pipelines so that immune cell findings become reproducible across cohorts? Therapeutically, the work here suggests that timing and selectivity will matter as much as target identity, given inflammation's dual role.

We thank the authors and reviewers whose work made this Research Topic possible. Together these contributions advance a more precise and more cautious account of neuroimmune interaction in disease—one that we hope will guide the next generation of mechanistic and translational studies.
